# Draft genome sequence of 2′-deoxyuridine-degrading *Enterobacter asburiae* strain C3 isolated from rice leaf sheaths

**DOI:** 10.1128/mra.00922-26

**Published:** 2026-09-29

**Authors:** Haowei Hu, Hiroka Maeshima, Aiko Tanaka, Sotaro Chiba, Ikuo Sato, Daigo Takemoto

**Affiliations:** 1 Graduate School of Bioagricultural Sciences, Nagoya University, Chikusa, Nagoya, Japan; USDA-ARS San Joaquin Valley Agricultural Sciences Center, Parlier, California, USA

**Keywords:** 2′-deoxyuridine, *Enterobacter asburiae*, rice blast

## Abstract

*Enterobacter asburiae* strain C3, isolated from rice leaf sheaths, degrades 2′-deoxyuridine, an infection-promoting metabolite produced by the rice blast fungus *Pyricularia oryzae*. Its 4.78-Mb draft genome comprises 24 contigs and complements functional studies of 2′-deoxyuridine degradation by strain C3.

## ANNOUNCEMENT

*Pyricularia oryzae* (syn. *Magnaporthe oryzae*) causes rice blast, a major disease of rice worldwide (1). The fungus produces 2′-deoxyuridine (dU), an extracellular metabolite that promotes infection in rice (2, 3). To identify dU-degrading bacteria, environmental samples were enriched in minimal medium containing dU as the sole carbon source. Strain C3 was obtained as a pure culture in 2023 from a mixed enrichment culture established from rice leaf sheaths collected in Mie Prefecture, Japan. The isolation and screening were described previously (3). For whole-genome sequencing, strain C3 was cultured in Luria-Bertani broth at 28°C with shaking at 120 rpm for 15 h, and genomic DNA was extracted using the NucleoSpin Microbial DNA kit (Takara Bio, Japan). A library prepared with the MGIEasy Fast PCR-Free FS Library Prep Set (MGI, China; Cat. 940-000021-00) was sequenced on a DNBSEQ-G400RS platform using 2 × 150 bp paired-end reads. Adapter and quality trimming were performed with fastp v1.1.0 (4), using automatic paired-end adapter detection and default filtering parameters (qualified-base Phred score ≥ 15; maximum proportion of unqualified bases, 40%; maximum number of N bases, 5; minimum read length, 15 bp), yielding 83,045,498 reads. Read quality was assessed with FastQC v0.12.1 (5) and summarized with MultiQC v1.27 (6). Paired-end reads were assembled *de novo* using SPAdes v4.2.0 with the --isolate option (7). Contigs shorter than 500 bp were excluded, leaving 147 contigs, and assembly statistics were calculated using QUAST v5.3.0 (8). Trimmed reads were mapped back using minimap2 v2.30-r1287 (9) and SAMtools v1.22.1 (10) to estimate per-contig depth. Taxonomic profiling was performed with Kraken2 v2.17.1 (11) using the PlusPF 16-GB database (k2_pluspf_16_GB_20260226). The median depth of contigs ≥ 10 kb was 2,587.875×, and 20% (517.575×) was used as an empirical low-depth threshold because it lay in a clear gap between excluded low-depth contigs (≤355.316×) and retained chromosome-associated contigs (≥2,334.01×). In total, 123 contigs were excluded based on low depth and/or taxonomic discordance. Six high-depth contigs (3,936×–21,677×) were retained as unplaced after nucleotide similarity searches and MOB-suite v3.1.9 analysis (12); four matched *Enterobacter* chromosomes across 100% of their query lengths (99.4%–100% identity), whereas two contained plasmid-associated markers. Species-level identification was evaluated using FastANI (13) against 26 complete *Enterobacter* RefSeq assemblies derived from type material and phylogenomic analysis with GToTree v1.8.16 (14). C3 showed its highest average nucleotide identity to *Enterobacter asburiae* (96.87% to ATCC 35953T), and a phylogeny based on 170 Gammaproteobacteria single-copy markers placed C3 as sister to the *E. asburiae* clade containing ATCC 35953T and FDAARGOS_892 (branch support = 0.998), supporting assignment of C3 to *E. asburiae*. The deposited assembly comprised 4,776,439 bp in 24 contigs, with an N50 of 439,181 bp and a GC content of 55.7% (Table 1). CheckM2 v1.1.0, using proteins predicted with Prodigal v2.6.3, estimated genome completeness at 100.0% and contamination at 0.24% (15, 16). The deposited assembly was annotated with DFAST (17), identifying 4,525 protein-coding sequences.

**TABLE 1:** Summary of the draft genome assembly and annotation of *Enterobacter asburiae* strain C3

Parameter	Value
Draft genome assembly size (bp)	4,776,439
No. of contigs^*a*^	24
N50 (bp)	439,181
L50	3
Largest contig (bp)	1,558,469
Smallest contig (bp)	623
GC content (%)	55.7
No. of quality-filtered reads	83,045,498
Protein-coding sequences	4,525
Completeness (CheckM2, %)	100.0
Contamination (CheckM2, %)	0.24

^*a*^ The deposited assembly comprises 18 chromosome-associated contigs and 6 additional unplaced contigs totaling 141,167 bp.

## Data Availability

The genome sequence is available in DDBJ/ENA/GenBank under accession BAAKFX000000000 and raw reads under accession DRR932716 (BioProject PRJDB40495; BioSample SAMD01826427).
